# Long-Term Clinical Outcomes of Transalveolar Maxillary Sinus Floor Elevation with Rotatory Instruments: An 8-Year Follow-Up Prospective Clinical Study

**DOI:** 10.3390/jcm14020365

**Published:** 2025-01-09

**Authors:** Álvaro Jiménez-Guerra, Eugenio Velasco-Ortega, Nuno Matos-Garrido, Iván Ortiz-García, Jesús Moreno-Muñoz, Enrique Núñez-Márquez, José-Luis Rondón-Romero, Naresh Kewalramani, Ángel-Orión Salgado-Peralvo, Loreto Monsalve-Guil

**Affiliations:** 1 Comprehensive Dentistry for Adults and Gerodontology, Faculty of Dentistry, University of Seville, 41009 Seville, Spain; alopajanosas@hotmail.com (Á.J.-G.); evelasco@us.es (E.V.-O.); nunogarrido@orallagos.pt (N.M.-G.); je5us@hotmail.com (J.M.-M.); enrique_aracena@hotmail.com (E.N.-M.); jolurr001@hotmail.com (J.-L.R.-R.); lomonsalve@hotmail.com (L.M.-G.); 2 Department of Nursery and Stomatology, Rey Juan Carlos University, 28008 Madrid, Spain; k93.naresh@gmail.com

**Keywords:** bone regeneration, dental implants, maxillary sinus, osseointegration, sinus floor augmentation

## Abstract

**Background:** Transalveolar sinus floor elevation (TSFE) is a surgical technique for the placement of dental implants in patients with reduced height of the maxillary posterior alveolar bone. This study aims to demonstrate the clinical outcomes of TSFE using the minimal invasive sinus elevation (MISE) technique in partially and totally edentulous maxillary patients. **Methods:** This prospective clinical study followed STROBE guidelines. TSFE was performed using the MISE technique with the simultaneous placement of implants. Dental implants were loaded at 6 months. Maxillary vertical bone gain was measured by CBCT, and marginal bone loss was assessed by periapical radiographs. **Results:** Ninety-one patients, with a mean age of 62.1 ± 11.8 years, were treated with TSFE and the placement of 107 implants, with a mean follow-up of 96.2 ± 11.7 months. An increase of 4.3 ± 0.4 mm in bone height was achieved, with a dental implant cumulative survival rate of 97.2%. Peri-implantitis was observed in 9.3% of implants, and membrane perforation occurred in 7.7% of cases. Technical complications were noted in 5.5% of patients. **Conclusions:** Within the limitations of this clinical study, it can be concluded that the MISE technique is a successful protocol for the placement of implants in the posterior maxilla with reduced height of the alveolar ridge, with a rate of biological and prosthetic complications below 10% over an average follow-up period of 8 years.

## 1. Introduction

In recent decades, dental implants have become the first choice in the rehabilitation of partially or edentulous patients, with predictable long-term results [1,2]. The placement of implants in the posterior edentulous area of the maxilla is conditioned by the presence of the maxillary sinus. This anatomical structure is lined by a very thin mucosa attached to the underlying bone, called Schneider’s membrane. After the loss of antral teeth, mainly molars and sometimes premolars, atrophy of the residual alveolar ridge occurs at the same time as the maxillary sinus increases in size, i.e., pneumatises, due to (1) the increased osteoclastic activity of the periosteum of Schneider’s membrane and (2) a positive increase in intra-antral pressure [3].

This situation makes implant therapy more difficult, requiring specific procedures such as the placement of short implants [4], while, in advanced cases of maxillary atrophy, the residual bone is insufficient to support dental implants, making it necessary to perform sinus floor elevation to achieve an adequate volume for their placement [2,5,6]. The residual volume of the bone, crest morphology, and available space for the prosthesis affect the treatment plan [7]. The literature shows that implants placed immediately after bone augmentation have slightly lower survival rates compared to those placed in native bone [8,9]. Several causal mechanisms have been proposed; bone substitutes often have a bradytrophic bone mechanism [10] and a lower quality of bed obtained after augmentation.

Sinus lift is based on the principle of separating Schneider’s membrane from the floor of the maxillary sinus, using it as a natural barrier membrane to create more bone volume for the simultaneous or delayed placement of dental implants [11]. Several surgical techniques have been described for sinus floor elevation since their introduction by Tatum in 1986. The lateral approach involves creating an osteotomy window to access the sinus (antrostomy) through the vestibular cortex. Subsequently, the Schneiderian membrane is elevated, and a filling material or bone graft is introduced to maintain the space for the placement of dental implants [12,13,14,15]. In 1994, Summers [16] described a transalveolar sinus floor elevation (TSFE) approach to the maxillary sinus, using osteotome instruments with progressive diameters. This technique increases the density of the maxillary bone through compaction, allowing the placement of implants with good primary stability and minimal trauma [17,18,19]. TSFE is considered less invasive than the lateral window approach, thus reducing postoperative complications and morbidity for the patient. On the other hand, it is less time-consuming and allows simultaneous implant placement, reducing the overall treatment time to functional prosthetic loading [20]. Furthermore, a recent systematic review [4] demonstrated higher implant survival rates for implants placed in sinuses regenerated by the TSFE compared to the lateral approach (95.4% to 100% vs. 75.57% to 100%, respectively). However, it may have limitations regarding the height achieved relative to residual bone height. If the residual alveolar height is 3–6 mm, TSFE has been shown to cause fewer complications [21,22]. However, some authors proposed the use of short implants (≤8 mm) in TSFE [23], as well as staged TSFE approaches [24] in order to perform transcrestal approaches in cases of minimal remaining bone heights. Another limitation is that Schneider’s membrane perforation may go unnoticed by the clinician as it is not directly visualized, making diagnosis and repair difficult. Furthermore, many complications, like postoperative headaches, vertigo, and inner ear injuries were associated with the classic technique [20].

Therefore, in order to reduce trauma and the risk of membrane perforation, a number of novel techniques have been introduced for TSFE with favorable results. The techniques investigated include the use of osteotomes, rotating instruments, a combination of osteotomes and trephine burs, and mechanical (hydraulic or by means of inflatable devices, such as the balloon sinus lift) pressure for detaching the endosinusal soft tissues from the sinus, as well as the use of counterclockwise rotating drills (such as Densah^®^ burs, Versah, Jackson, MI, USA), secondarily increasing the bone quality of the dental implant site. In addition, techniques have been described that fracture the cortex of the sinus floor, as well as others that perforate the sinus floor without fracturing it [24]. Ultrasonic piezoelectric instruments are designed to work on the bone without perforating the Schneiderian membrane and cut mineralized structures without disturbing soft tissues at the sinus floor. Additionally, these surgical techniques can include the use (or not) of graft biomaterials [25,26,27]. The treatment of choice for rehabilitation of the atrophic posterior maxilla is influenced by several factors [28]. The type of approach to be used depends on the height [28,29] and/or width of the remaining alveolar ridge, the intrasinusal anatomy, the number of teeth to be replaced [29] and the possibility of achieving sufficient primary implant stability [28], although other factors, such as the operator’s surgical experience and personal preferences, should also be considered [20,29].

Several systematic reviews indicate that TSFE represents a valid option for subantral bone augmentation and implant survival rates [4,25,30]. In this regard, the minimal invasive sinus elevation (MISE) technique has been introduced to perform TSFE safely and atraumatically. This technique employs rotating and calibrated milling instruments—by means of drill depth stops—of different lengths to achieve precise preparation of the implant bed [31], rather than compressing or fracturing the remaining bone volume. These drills allow the Schneider membrane to be raised gradually (1 mm at a time), preventing perforation. Specifically, an initial opening is created using cylindrical drills, followed by a chamfered, flat-tipped drill, which deforms the floor of the sinus and perforates it if it is thin enough. Otherwise, break-up drills are used. Finally, rounded drills, which are round-tipped and non-cutting, smooth the dental implant bed, and increase the diameter according to the diameter of the implant to be placed. An advantage of this technique is that can be successfully adopted even with a residual bone height lower than 6 mm [32].

Since its introduction in the early 2000s, little research has focused on the analysis of its long-term clinical outcomes. Therefore, this study aimed to analyze the long-term (8-year follow-up) clinical and radiographic outcomes of the MISE technique, in terms of bone changes and complications, in the treatment of totally and partially edentulous maxillary patients.

## 2. Materials and Methods

This prospective clinical study was conducted at the master’s degree clinics of the Implant Dentistry course at the Faculty of Dentistry, University of Seville (Seville, Spain), from December 2011 to November 2014. The study followed STROBE (Strengthening the Reporting of Observational Studies in Epidemiology) guidelines [33]. All procedures performed in this study were in accordance with the ethical standards of the institutional and/or national research committee and with the 1964 Helsinki Declaration and its later amendments or comparable ethical standards. The research protocol was approved by the ethics committee of the University of Seville (9 September 2011).

### 2.1. Participants

All volunteers received clear and honest information about the nature and objectives of the study before testing. Written consent was obtained from all participants.

#### 2.1.1. Inclusion Criteria

Included patients were of both genders, aged over 18 years, who were fully or partially edentulous, classified as type I or II patients according to the American Society of Anesthesiologists (ASA) classification, and smoked < 10 cigarettes per day, with a need for TSFE, and with a preoperative height of the available alveolar bone ≥ 4 mm.

#### 2.1.2. Exclusion Criteria

Excluded patients were those with severe systemic disease (ASA type III or IV), untreated or uncontrolled periodontal disease, plaque index scores ≤ 20% [34], coagulation disorders, bruxism, pregnant women, those with medical disorders related to altered bone metabolism, and those who were not maintaining their dental implants. Also excluded were patients undergoing current radiotherapy or chemotherapy treatment of the head and/or neck, or who had undergone such treatment less than two years ago, immunosuppressed patients, those with alcohol or drug abuse, and patients who smoked ≥ 10 cigarettes per day.

### 2.2. Clinical Protocol

Treatment planning included an oral examination, cone beam computerized tomography (CBCT) (Pointnix 800 HD 3D plus^®^, Seoul, Republic of Korea) (FOV = 12 × 10 cm; initial = 9 mA 80 kVp—final = 10 mA 80 kVp), diagnostic casts for intermaxillary relations, and clinical photographs. Patients were informed of all possible implant treatments and accepted the clinical protocol.

Before surgery, the patients received preventive antibiotic therapy [35], i.e., amoxicillin/clavulanic acid 500/125 mg, administered 1 h before surgery, and continued taking 3 capsules daily for 7 days and, in patients sensitive to the use of ß-lactams, ciprofloxacin 500 mg/12 h, starting 1 h before surgery, was prescribed [36,37]. Postoperatively, a 0.20% chlorhexidine digluconate mouthwash (PerioAid^®^ treatment, Dentaid, Barcelona, Spain) was prescribed twice daily for 15 days. Ibuprofen (600 mg, 4 times daily) was prescribed for 7 days. All patients were treated under local anesthesia using articaine with adrenaline.

The TSFE was performed using the MISE system (Maxillary Indirect Sinus Elevation^®^, Sweden-Martina^TM^, Padua, Italy) which consists of a system of drills and stops that allows the maxillary sinus to be raised atraumatically and gradually to a height of 5–10 mm above the initial situation [31,32]. The elevation is gradual and predictable (progression of 1 mm at a time), preserving the Schneiderian membrane and allowing the introduction of the filling material. The preparation of the bed and the placement of the implants were carried out according to the standardized protocol, using the drills with stops at a constant speed of 800 revolutions per minute (RPM). The Valsalva maneuver was performed to assess the integrity of the Schneiderian membrane [38].

Two surgical techniques for the placement of implants were established: the submerged technique (two surgeries) and the non-submerged technique (one surgery). Several types of dental implants were placed: IPX^®^ (Galimplant^TM^, Sarria, Spain) with a sandblasted and etched surface and internal hexagonal connection; Premium Kohno^®^ (Sweden-Martina^TM^, Padua, Italy) with internal hexagonal connection and a surface treated by sandblasting with zirconium oxide and etching with acids; Osseotite^®^ (Biomet 3i^TM^, Palm Beach Gardens, FL, USA) with an external connection and a surface treated with double acid etching; and Tapered Self Thread^®^ (Hi-Tec^TM^, Herzlia, Israel) with an internal connection and a hydroxyapatite-coated surface.

The selection of the number, length, and diameter of the implants depended on the volume and quality of the residual bone, as well as the treatment plan for TSFE. The bone substitutes used for maxillary augmentation were: Spongostan^®^ porcine collagen (Ferrosan^TM^, Soeborg, Sweden), Osteoblast^®^ ß-tricalcium phosphate (β-TCP) (Galimplant^TM^, Sarria, Spain), and Ladec^®^ mineralized bovine bone (Biohorizons^TM^, Birmingham, AL, USA).

### 2.3. Prosthetic Protocol

In cases where the submerged technique was used, second-stage surgery was performed at 4.5 months, and impressions were taken 15 days later. In the case of the non-submerged technique, impressions were taken 5 months after implantation. A conventional loading protocol was established with the placement of the corresponding fixed prosthesis 6 months after the placement of the implants.

### 2.4. Follow-Up

**Figure 1:**
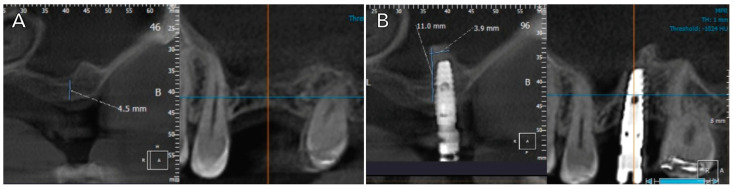
(**A**) Preoperative CBCT; (**B**) post-operative CBCT (5-month follow-up before impression taking).

**Figure 2:**
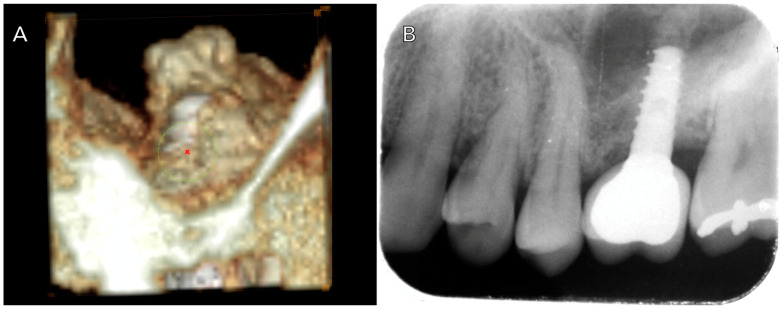
(**A**) Intrasinusal image showing bone formation at the expense of the sinus at 5 months postoperatively; (**B**) periapical radiograph at 4 years follow-up.

The elapsed time of clinical follow-up since the prosthodontic rehabilitation was at least 80 months. Several conditions, including dental implant stability, an absence of radiolucency around the implants, mucosal suppuration, and pain, were used for the assessment of implant survival. Follow-up visits were scheduled at 3 and 6 months after dental implant placement, and annually thereafter, following the placement of the prostheses. Marginal bone loss (MBL) was evaluated based on digital periapical radiographs taken perpendicular to the long axis of the implants, comparing the differences between the 1-year follow-up radiographs and the 8-year follow-up radiographs. Changes in bone height were assessed by comparing the CBCT scans acquired before treatment and after surgery (at 4 years and 8 years of the study) (Figure 1 and Figure 2).

Furthermore, demographic and clinical data included patient information (gender, age, dental health, history of periodontitis, systemic diseases, smoking habits), details about the placed implants (type, number, position, diameter, and length), and the prosthetic rehabilitation, including the dates of delivery. Additionally, the analyzed data included all information about any implant failure or biological and technical complications that occurred during the intervention, after surgery and functional loading, and at each follow-up visit.

### 2.5. Statistical Analyses

All data from the study were analyzed using the SPSS software package (version 11.5.0, SPSS, Chicago, IL, USA). Descriptive statistics were utilized to report the general results of the study. For all qualitative variables, values were expressed in absolute terms and percentages (%), and the chi-square test was used for calculations. For quantitative variables, the means, standard deviations (SD), medians, ranges, and 95% confidence intervals (CI) were calculated. Group similarities were confirmed using analysis of variance (ANOVA). The Mann–Whitney U non-parametric test was used to compare differences between groups based on various risk factors. To determine the relationship between MBL and different variables, the Mann–Whitney U test was used for dichotomous variables, and the Kruskal–Wallis test was used for variables with more than two categories. A *p*-value < 0.05 was considered statistically significant.

## 3. Results

### 3.1. Characteristics of the Patients and Dental Implants Placed

The mean follow-up period was 96.2 ± 11.7 months, ranging from 80 to 195 months. The study included 91 partially edentulous maxillary patients, comprising 49 females and 42 males, with ages ranging from 32 to 87 years (mean age: 62.1 ± 11.8 years), who were treated using the MISE technique. A total of 107 implants were placed simultaneously. Statistical analysis revealed no significant differences related to sex and age (*p* = 0.1346). Fourteen patients (15.4%) had a prior history of periodontitis. Twenty-one patients (23.1%) were smokers, and twenty-four patients (26.4%) had a systemic disease (e.g., diabetes, hypertension) (Table 1).

Among the 107 implants placed, 102 (95.3%) had a diameter of 4 mm, while 5 implants (4.7%) had a diameter of 5 mm. In terms of length, 55 implants (51.4%) were 10 mm, 34 (31.8%) were 12 mm, 16 (14.9%) were 11.5 mm, and 2 (1.9%) were 5.8 mm. Three implants were lost during treatment, resulting in a cumulative survival rate of 97.2% (Table 2).

### 3.2. Biomaterials Used

Biomaterials were not used in two patients (2.2%). Porcine collagen was used in 38 patients (41.8%), porcine collagen combined with β-TCP was used in 28 patients (30.8%), porcine collagen combined with mineralized bovine bone was used in 12 patients (13.2%), and β-TCP alone was used in 11 patients (12.1%) (Table 2).

### 3.3. Mean Bone Changes

The preoperative height of the available alveolar bone was 6.3 ± 1.35 mm. An increase in bone height was observed postoperatively (4.3 ± 0.4 mm), with an overall bone height measured at 8 years after the initial surgery being 10.6 ± 0.9 mm. Measurements of bone height according to tooth positions revealed no significant difference between premolars and molars.

The mean MBL was −1.16 ± −0.72 mm, ranging from −0.5 to −2.6 mm during the follow-up period. There were no significant differences in MBL when correlated with demographic and clinical variables (Table 3).

### 3.4. Characteristics of the Implant-Supported Prosthesis

Regarding the types of prostheses, single crowns were placed in 44 patients (48.4%), fixed bridges in 44 patients (48.4%), and ball overdentures in 3 patients (3.2%). Screw-retained prostheses, including those with attachments for ball overdentures, were performed in 58 patients (63.7%), while cement-retained prostheses were used in 33 patients (36.3%).

### 3.5. Biological and Mechanical Prosthodontic Complications

The most frequently reported intraoperative complication was perforation of the sinus membrane, occurring in seven patients (7.7%). During the follow-up period, nine patients (9.9%) experienced postoperative complications, with three patients (3.3%) reporting loss of implants and six patients (6.6%) experiencing prosthetic complications, such as loosening of the prosthetic connection screws (n = 5 patients; 5.5%) and debonding of the fixed prosthesis (n = one patient; 1.1%). Peri-implantitis was identified in 10 implants (9.3%) in eight patients (8.8%), with a higher prevalence among smokers (62.5%) (Table 4).

## 4. Discussion

The present study reports the clinical findings of treating the posterior maxilla with implants placed using the MISE technique. The clinical and radiographic results demonstrate a high success rate (97.2%) for the implants placed with this technique, with a mean bone gain of 4.3 mm over a follow-up period of 8 years. During the entire clinical follow-up period, three implants failed. This underscores the clinical importance of this prospective study, highlighting that the MISE technique represents a predictable and safe method for elevating the floor of the maxillary sinus.

These results have been corroborated by several studies [26,39,40,41]. A retrospective study reported the clinical and radiographic outcomes of TSFE using a hydraulic device over 4 years. One hundred and thirty-six TSFE procedures were performed on 110 patients, with a mean follow-up period of 48 months. The 4-year dental implant survival rate was 97% (n = 196/202), with six early implant losses. Additionally, 96.4% of patients reported either no or minimal discomfort [41]. Another retrospective study analyzed the efficacy of flapless TSFE. Seventy-one elevations with simultaneous implant placement were performed on fifty-two consecutive patients over a mean period of thirty months. Following an initial pilot bur transmucosal perforation, progressively larger osteotomes were used. The cumulative success rate during the observation period was 95% [26].

A 5-year clinical study assessed the outcomes of TSFE in 26 patients, in whom 30 implants were placed, achieving a 100% dental implant survival rate [40]. Another clinical study reported the results of a modified osteotome TSFE in cases with residual bone height less than 5 mm. Thirty patients were treated (18 patients with < 5 mm and 12 with ≥ 5 mm). The dental implant survival rate was 100%, and after 6 months, the height of the graft apically between the two implants gradually stabilized at 8.92 mm. There was no significant difference in graft bone resorption between patients with < 5 mm and those with ≥ 5 mm of residual bone height [39].

Residual bone height is a crucial determining factor in the survival rate of implants placed using the TSFE technique [42,43]. The prognosis varies depending on whether the residual height is < 5 mm or ≥ 5 mm. Several studies recommend that the existing vertical bone dimension at the implant site should be at least 4–6 mm [42,43,44]. A recent study reported the use of the TSFE technique in 72 patients, with 102 implants placed using osseodensification drills. This study demonstrated that the technique appears to be a fast, effective, and safe method. Osseodensification drills compact and lateralize the bone, potentially increasing the initial mechanical stability around the simultaneously placed implants [45].

In the present research study, the mean remaining bone height of 6.28 mm increased by 4.3 mm, and more than 95% of the implants placed had a length between 10 and 12 mm. After a mean clinical follow-up period of 96.2 months, the radiological study has demonstrated the presence of newly formed bone around the portion of the implants introduced into the maxillary sinus. Scientific evidence has shown that several techniques proposed for TSFE have resulted in bone formation around the implant body in the bony sinus cavity of patients after the elevation to the desired height [21,46,47,48]. These results align with those published in a systematic review and meta-analysis [47] (2023), which reported an endosinusal bone increase of 3.12 to 5.5 mm when biomaterials were used, and 1.9 to 3.7 mm when they were not. The study established adequate bone gain values for this technique, ranging from 1.9 to 5.6 mm, which is believed to allow stress distribution in the tissues without negatively affecting their health.

Histologic assessment reported osteoclasts actively resorbing the graft as well as osteoblasts forming new bone. In the severely atrophic maxilla, the use of bone substitutes promotes new bone formation while being slowly absorbed [46]. After an initial period of three months, a bone regeneration process appears to take place, inducing the migration, adhesion, and proliferation of osteoblasts inside the graft, and promoting angiogenesis [48]. The bone remodeling process takes more than three months to repair the damage caused by conventional drills, which remove a significant amount of bone, and cause strains in the walls of osteotomies that reach or exceed the bone microdamage threshold. Therefore, this surgical technique helps preserve bone and increase density. Additionally, the healing process creates compressive forces against the implant, thus enhancing bone-to-implant contact, which has been shown to promote osteogenic activity and successful osseointegration [21].

In the present research study, various bone substitutes, such as porcine collagen, ß-TCP, and mineralized bovine bone were used, either alone or in combination. With the use of these biomaterials, an implant success rate of 97.2% and a mean bone gain of 4.3 mm were obtained after a mean follow-up period of 8 years. Various studies of TSFE have demonstrated clinical and radiological success through the use of biomaterials to seal the newly formed space [21,39,41,42,45]. However, the simultaneous use of grafts for TSFE remains a subject of controversy [49,50,51,52,53,54]. The biological principles on which the technique elevation of the maxillary sinus are based are fundamentally based on the osteogenesis that develops when the elevation of Schneider’s membrane is performed, and the space formed is filled with a blood clot. It is believed that this osteogenesis is induced by the stimulation of progenitor cells from the periosteum, or that the membrane itself has an osteogenic potential that would lead to the formation of new bone. The biological principles underlying the elevation of the maxillary sinus are fundamentally based on the osteogenesis that occurs when Schneider’s membrane is elevated, and the space formed is filled with a blood clot. It is believed that this osteogenesis is induced by the stimulation of progenitor cells from the periosteum or that the membrane itself has an osteogenic potential that would lead to the formation of new bone [52,53]. These biological considerations may explain the clinical efficacy of TSFE, both with and without the use of bone grafts or substitutes. Several studies and systematic reviews indicate the high success of this approach, generally finding no significant differences in clinical outcomes, and postulate that bone substitutes are not essential for achieving success with this surgical technique [49,50,51,52,53,54].

MBL is an important criterion for the success of implant therapy. In the present study, after an 8-year follow-up period, the MBL was −1.16 ± −0.72 mm. Several clinical studies of TSFE have reported MBL of implants [21,27,40,54,55]. At 5 years, an average MBL of −0.30 ± −0.11 mm (SD= −0.12 to −0.52) has been observed [40], while another study reported a MBL of 1.98 mm at the same follow-up periods [54].

As clinical follow-up time increases, MBL also tends to increase. In a long-term research study (18-year follow-up), an MBL of −2.1 ± −0.9 mm (SD = −0.3 to −4.0 mm) at the mesial, and −2.1 ± −0.7 mm at the distal aspect (SD = −1.2 to −3.6 mm) has been reported [22].

The incidence of complications in the TSFE technique is low. After surgery, some cases of pain, swelling, benign paroxysmal vertigo, and membrane perforation have been described, which have resolved spontaneously [25,54]. Low to moderate postoperative pain and swelling have been reported in some studies, without clinical relevance [45,54,56]. However, some clinical research on TSFE has reported no complications or adverse effects among treated patients, with an overall success rate of 100% [27,39,56]. The most frequent intraoperative complication has been perforation of the sinus membrane [25,41,54,57,58]. A retrospective multicenter study performed on clinical and radiographic records of patients who underwent TSFE reported membrane perforation in 7.2% of 418 treated patients [58]. In the present study, only seven patients (7.7%) reported membrane perforation, which was not related to dental implant loss [59]. A recent systematic review [60] observed a significantly increased association between Schneider membrane thickness and increased risk of perforation; however, the meta-analysis failed to demonstrate significant differences between the cut-offs of membrane thickness levels of 2 mm, 1.5 mm, and 1 mm. In this regard, one study found a higher prevalence of perforations in excessively thin or excessively thick membranes, i.e., ≤ 0.5 mm or > 3 mm (17% and 25%, respectively), with the lowest prevalence described (7%) in those 1–1.5 mm thick [61].

Another biological complication reported was peri-implantitis, with a prevalence of 9.3% at the implant level and 8.8% at the patient level. This was higher among smokers (62.5%), who accounted for 23.1% of the sample. Its higher prevalence in these patients has been widely demonstrated in the literature. In this regard, a recent systematic review [62] (2023) put the relative risk of peri-implantitis among smokers at 2.04 (95% CI = 1.46–1.85) at implant level and 2.79 (95% CI = 1.42–5.50) at patient level. A recent systematic review and meta-analysis [60] (2022) reported a higher prevalence of peri-implantitis compared to the present study, specifically 19.53% (95% CI, 12.87–26.19%) at the patient level and 12.53% (11.67–13.39%) at the implant level, with no significant differences between studies with 5–9 years of follow-up and studies with greater longevity. The consensus report of the World Workshop on the Classification of Periodontal and Peri-Implant Diseases and Conditions (2017) [61] recommended excluding probing depth as a diagnostic criterion. In the present study, these recommendations were followed, whereas studies that included probing depth reported higher figures, both at the patient level (17.56% vs. 24.69%, respectively) and at the implant level (11.99% vs. 15.21%, respectively), although the differences were not statistically significant (*p* = 0.27 and *p* = 0.31, respectively).

Clinical outcomes of TSFE report the incidence of technical complications [22,32]. Sixteen patients treated with the MISE technique followed by a mean implant loading period of 17.5 years reported small chipping within the ceramic veneering in 42.8% of crowns. All the chippings were polished, and replacement of the reconstruction was not needed. One case of screw loosening was reported and resolved within a single appointment [22]. A study assessed 17 edentulous patients who received 20 implants and sinus floor elevation. After 24 months, one abutment on a single implant was observed unscrewed before the last follow-up. The technical complication required the removal of the crown, rescrewing of the implant/abutment screw, and adjustment of occlusal contacts [32]. In the present study, five patients (5.5%) reported the loosening of connection screws (4.4%) and debonding of the fixed prosthesis (1.1%). These prosthetic complications have been associated with occlusal overload and it is recommended that the patient’s occlusion and idiosyncratic prosthetic factors be assessed beforehand and periodically re-evaluated to reduce their rate of occurrence.

### 4.1. Strengths and Limitations

The main strength of the present study is its long follow-up period, as well as the CBCT evaluation of bone level changes.

One of the main limitations of the present study was the non-registration of Schneider’s membrane thickness to determine its possible influence on the prevalence of perforations beyond the surgical technique itself, as well as the heterogeneity of the variables included in the study. In this regard, various subsinusal cavity-filling biomaterials, different dental implant systems, as well as different types of prostheses, both removable and fixed, were used in the rehabilitation of the patients. Nevertheless, this variability contributes to an overview of the response of implants placed through TSFE using the MISE technique.

### 4.2. Recommendations for Further Research

Future studies should aim to investigate the clinical and radiographic results of the MISE technique through randomized studies using the same dental implant system and the same biomaterials to rule out possible confounding factors. Along the same lines, it is recommended that the thickness of the Schneider’s membrane be recorded beforehand by CBCT.

## 5. Conclusions

Despite the limitations of the present clinical study, it can be concluded that the MISE technique is a safe and predictable surgical technique for the placement of dental implants in the posterior area of the maxilla, with reduced height of the alveolar ridge. The average bone height increase was 4.3 ± 0.4 mm, and the simultaneous implant placement survival rate was 97.2%. The most frequent intraoperative complication was Schneider’s membrane perforation. Additionally, the most frequent biological and mechanical complications were peri-implantitis and loosening of prosthetic screws, respectively. However, these were lower than the 10% over an average follow-up period of 8 years, indicating a successful dental implant protocol with long-term favorable outcomes.

## Figures and Tables

**Table 1:** Description of patient’s characteristics.

Variable	Specifications	N ^1^	% ^2^
Participants		91	100
Gender	Male	42	46.1
	Female	49	53.9
History of periodontitis		14	15.4
Smokers		21	23.1
Systemic diseases		24	26.4

^1^ Number of patients; ^2^ percentage.

**Table 2:** Description of dental implant characteristics and biomaterials used.

Variable	Specifications	N ^1^	% ^2^
Implants placed		107	100
Implant diameter	4 mm	102	95.3
	5 mm	5	4.7
Implant length	5.8 mm	2	1.9
	10 mm	55	51.4
	11.5 mm	16	14.9
	12 mm	34	31.8
Biomaterials	None	2	2.2
	Porcine collagen	38	41.8
	Porcine collagen + β-TCP ^3^	28	30.8
	Porcine collagen + mineralized bovine bone	12	13.2
	β-TCP	11	12.1

^1^ Number of patients; ^2^ percentage; ^3^ β-tricalcium phosphate.

**Table 3:** Correlation between MBL and clinical variables.

Variable	Specifications	MBL ^1^ (mm)	*p*-Value
Age	≤54 years	1.14 ± 0.71	0.6798
	55–64 years	1.26 ± 0.67	
	≥65 years	1.08 ± 0.72	
	Total	1.16 ± 0.72	
Gender	Male	1.17 ± 0.79	0.8386
	Female	1.15 ± 0.71	
	Total	1.16 ± 0.72	
History of periodontitis	Yes	1.16 ± 0.68	0.8154
	No	1.15 ± 0.80	
	Total	1.16 ± 0.72	
Smokers	Yes	1.18 ± 0.79	0.8570
	No	1.15 ± 0.67	
	Total	1.16 ± 0.72	
Medical history	Yes	1.04 ± 0.55	0.3626
	No	1.12 ± 0.74	
	Total	1.16 ± 0.72	
Implant system	Osseotite^®^ (Biomet 3i^TM^, Palm Beach Gardens, FL, USA)	1.22 ± 0.64	0.4479
	IPX^®^ (Galimplant^TM^, Sarria, Spain)	1.15 ± 0.72	
	Tapered Self Thread^®^ (Hi-Tec^TM^, Herzlia, Israel)	1.15 ± 0.74	
	Premium Kohno^®^ (Sweden-Martina^TM^, Padua, Italy)	1.15 ± 0.73	
Implant diameter	4 mm	1.16 ± 0.69	0.6700
	5 mm	1.10 ± 0.93	
	Total	1.16 ± 0.72	
Implant length	8.5 mm	0.50 ± 0.43	0.7686
	10 mm	1.15 ± 0.73	
	11.5 m	1.24 ± 0.66	
	12 mm	1.18 ± 0.63	
Prostheses	Single crowns	1.10 ± 0.67	0.5385
	Fixed bridges	1.19 ± 0.70	
	Ball overdentures	1.26 ± 0.64	
	Total	1.16 ± 0.72	
Follow-up	≤96 months	1.13 ± 0.64	0.6421
	>96 months	1.20 ± 0.77	
	Total	1.16 ± 0.72	

^1^ Marginal bone loss.

**Table 4:** Description of patients with complications.

Variable	Specifications	N ^1^	% ^2^
Schneiderian membrane perforation		7	7.7
Postoperative complications		8	8.8
Dental implant loss		3	3.3
Biologic complications (peri-implantitis)	Patient level	8	8.8
	Dental implant level	10	9.3
	Smokers	5	62.5
Prosthetic complications	Loosening	5	5.5
	Debonding of the fixed prosthesis	1	1.1
Mean MBL ^3^ = 1.16 ± 0.72 mm (0.5 to 2.6 mm)			

^1^ Number of patients; ^2^ percentage; ^3^ marginal bone loss.

## Data Availability

The datasets generated during and/or analyzed during the current study are available from the corresponding author on reasonable request.

## Abbreviations

TSFE—transalveolar sinus floor elevation; MISE—minimal invasive sinus elevation; STROBE—Strengthening the Reporting of Observational Studies in Epidemiology; ASA—American Society of Anesthesiologists; CBCT—cone beam computerized tomography; RPM—revolutions per minute; β-TCP—β-tricalcium phosphate; MBL—marginal bone loss; SD—standard deviation; CI—confidence interval.
